# Plea for a Simple But Radical Change in Scientific Publication: To Improve Openness, Reliability, and Reproducibility, Let’s Deposit and Validate Our Results before Writing Articles

**DOI:** 10.1523/ENEURO.0318-22.2022

**Published:** 2022-09-13

**Authors:** Jean-Antoine Girault

**Affiliations:** 1 Institut National de la Santé et de la Recherche Médicale UMR-S 1270, 75005 Paris, France; 2 Faculty of Sciences and Engineering, Sorbonne University, 75005 Paris, France; 3 Institut du Fer à Moulin, 75005 Paris, France

## Abstract

Limited reproducibility and validity are major sources of concern in biology and other fields of science. Their origins have been extensively described and include material variability, incomplete materials and methods report, results selection, defective experimental design, lack of power, inappropriate statistics, overinterpretation, and reluctance to publish negative results. Promoting complete and accurate communication of positive and negative results is a major objective. Multiple steps in this direction are taken, but they are not sufficient and the general construction of articles has not been questioned. I propose here a simple change with a potentially strong positive impact. First, when they complete a substantial coherent set of experiments, scientists deposit their positive or negative results in a database [“deposited results,” (DRs)], including detailed materials, methods, raw data, analysis, and processed results. The DRs are technically reviewed and validated as “validated DRs” (vDRs) or rejected until satisfactory. vDR databases are open (after an embargo period if requested by the authors) and can later be updated by them or others with replications or replication failures, providing a comprehensive active log of scientific data. Articles, in this proposal, are then built as they currently are, except they only include vDRs as strong and open building blocks. I argue that this approach would increase the transparency, reproducibility, and reliability of scientific publications and have additional advantages including accurate author credit, better material for evaluation, exhaustive scientific archiving, and increased openness of life science material.

## Significance Statement

Publication validity and reproducibility are major issues in life sciences with an important cost, decreased research yield, and slowed progress. A number of initiatives are currently underway but they are partial or insufficient and do not challenge the way scientific material is made publicly available. I propose a radical but in essence simple change to address these limitations, a two-step process made possible by current information technology. First, deposit in dedicated databases and validation of comprehensive information on coherent sets of completed experiments. Second, preparation and submission of articles using only these deposited and validated results. This would improve reliability and reproducibility, but also address a number of additional issues including openness, credit, evaluation, and archiving.

## Introduction

The validity of many results obtained in biological sciences, medicine, and social sciences has been repeatedly questioned because of their poor reproducibility (Ioannidis, 2005; Prinz et al., 2011; Camerer et al., 2018). This is a major source of concern for scientists, journals, funding bodies, and industry. The general public is also alerted since this subject is relayed by the media, without the nuances necessary for its proper understanding. It can raise doubts about the scientific approach and fuel antiscience trends (Achenbach, 2015). Despite these limitations, biology moves forward at a steady pace, but poor reproducibility and limited validity are likely to decrease the yield of research, slowing progress and increasing cost (Freedman et al., 2015). Ways to improve the situation have been put forward, and journals have started requesting better statistical analyses, more raw data, and detailed methods, a posteriori. Yet the way articles are constructed has not been questioned. I propose a radical change, which is now made possible by current information technology and which would have a profound impact on reproducibility and validity and would make science fully open. The principle is very simple: first, when scientists complete a coherent group of experiments, they deposit their full methods and results in a “validating” database, which is open (after an embargo period if requested) and updatable. Second, later to write articles scientists use as many sets of validated deposited results (DRs; already open or still under embargo) as needed. Conclusions and hypotheses in published articles are thus based on strong, open, and updatable data.

## Sources of validity and reproducibility failures

The sources of the reproducibility and validity failures are multiple and have been thoroughly described (Ioannidis, 2005; Prinz et al., 2011; Ioannidis, 2014; Munafò et al., 2017; Nimpf and Keays, 2020). In biology, difficulties are inherent to the variable nature of the object under study: results can differ depending on cell lines, clones, animal species, strains, sex, age, circadian or seasonal variations, or the way animals are housed. There is also variability in biological, or even chemical, reagents, including between providers, batches, and storage or preparation procedures. Success of delicate experiments may depend on a performer’s dexterity and/or tiny details. Many of these differences are currently impossible to track because materials and methods are not described in sufficient detail. Even when they are carefully recorded in laboratory notebooks, the information remains confidential, inaccessible to the scientific community, and can be lost.

Problems may also arise from design, analyses, and interpretation ranging from flawed experimental design or underpowered experiments to inadequate statistical analysis and overinterpretation of data (Ioannidis, 2014). Hidden aspects complicate the problem, such as exclusion of “aberrant” data points (outliers) or “failed” experiments. Although exclusion is clearly justified when external interference, such as a sick animal or a defective reagent, is clearly identified, the boundaries are ill defined and the rationale should not be hidden. Outliers and failed experiments should, I argue, be part of the report but not included in the final analyses with explicit justification of their exclusion. Despite a general effort to improve all these issues, it is difficult to estimate the extent of their persistence, presumably still high (Yarborough et al., 2019).

As often pointed out, “publish or perish” is part of the problem, as well as the reluctance of authors and journals to publish negative or confirmatory results (Nimpf and Keays, 2020). Peer review is expected to detect and correct errors and shortcomings, but the process is far from being perfect. For example, because biological sciences are increasingly complex and technically sophisticated, questions are addressed with multiple approaches. “Big” papers contain a plethora of results with a nice storyline that we love reading (Sanes, 2019), as we love watching high-budget movies. But scientific papers are not fiction. Reviewers have limited time, and each of them masters only a few of the techniques used. A nice storyline and/or the potential high impact of the findings may render the authors and reviewers less critical about some experimental details “where the devil is.”

## Examples of current approaches to address the reproducibility crisis

Many excellent suggestions to improve the quality of published science have been made, while acknowledging the difficulties of their implementation (for example, see Ioannidis, 2014; Munafò et al., 2017; Yarborough et al., 2019). Recent evolutions have also brought significant improvement.

Unpublished preprint publication is important progress, whose amazing success demonstrates the possible speed of evolution in the way science is communicated. Preprint servers allow faster access to novel material and possible feedback from any reader before the work is formally submitted. Some journals, such as *eLife*, now accept direct submission and review of material already in *BioRxiv*, while others provide preprint publication service at the time of submission. These are welcome steps, which simplify the submission process. However, such preprints are just unreviewed article manuscripts with all the limitations of current articles that are discussed above. The advantages of preprint publication and the changes in articles composition proposed here (see below) will be easily combined with the publication on open databases of full articles comprising vDRs before they are actually submitted or accepted.

A different useful development is study preregistration that increases methodological and statistical rigor. It is not applicable to all types of research and does not prevent all the reporting insufficiencies described above.

Although a growing number of top journals encourage authors to make available more experimental details (e.g., a list of antibodies) and raw data, and are increasingly demanding about the quality of statistical analyses, these steps occur a posteriori, often late after experiments are completed, and rely mostly on the authors’ willingness for precision and exhaustiveness (a daunting task in manuscripts that include numerous experimental results).

Improvements are also underway with respect to publication of negative results, as illustrated by an increasing number of journals encouraging the publication of negative results (including *eNeuro*: https://www.eneuro.org/content/general-information#types).

It is important to stress that in areas of quantitative biology such as genomics, transcriptomics, or structural biology, the availability of full datasets is already the rule, not the exception. However, as in other domains, key details about the experiments from which the datasets are generated can be missing.

Finally, I would like to mention the recent and laudable initiative of “micropublication” (Raciti et al., 2018), which shares some features with the current proposal, such as open publication of simple data, but with important differences including the fact that in my proposal deposited results are not a final publication but the building block of full articles.

Thus, the recent progress does not address most of the issues and limitations that hinder high-quality science publication, but it clearly shows that the way scientists publish their results is improving and that further progress is possible and will be welcome if it is recognized as being useful.

## Proposal of a two-step procedure: results deposit and validation before article submission

Here, I propose to dissociate the publication of results with their detailed materials, methods, and analytical procedures, as DRs, from that of articles. The deposit and validation of results would be the first step in open scientific communication and would provide the strong material from which next-generation scientific articles will be build. Such a change was not feasible a few years ago because of obvious technical limitations, but it is now possible with current information technology and powerful databases. In this proposal, the unit of DRs corresponds to a specific experiment or group of highly related experiments (including replications if already conducted). Preliminary or first results cannot be considered as DRs, which must correspond to a full experiment or set of related experiments with clear and conclusive (positive or negative) results. At the other end of the spectrum, a very complex set of experiments and results that can be divided into logically and methodologically separated subsets, could be deposited as several DRs. To be deposited, a DR includes (1) a clear exposure of the precise biological question that the experiment directly aims to answer; (2) the minute details of all resources used (e.g., equipment location and description, reagent sources and batches, cell lines, or animals precise description); (3) the meticulous report of all procedures (step by step, mentioning possible procedure variations, including videos if necessary); (4) all raw data, processed data, processing procedures (including macros or software); and (5) final results (e.g., images, tables, plots, graphs) with their detailed statistical analyses. In brief, a DR contains all that is needed to assess the quality of the results and reproduce exactly the same experiments. It is very close to what laboratory notebooks (should) contain. It also includes required additional regulatory or ethical information regarding animal experiments or human material or participation. Outliers or failed experiments, if any, are indicated, and the rationale for their exclusion provided. In contrast, authors do not have to justify why they did the experiment, in what intellectual line of research it is included, or what the broad implications might be. DRs include just the facts, but all the facts needed to reproduce procedures and compare results. Results can be “positive” (an effect is observed) or “negative” (no effect is observed), but they have to be convincingly supported by the experimental evidence. The DR is reviewed by two experts in the methodological approach and statistics are checked. If any information is missing or flaws are detected, the authors are requested to revise and resubmit (these exchanges are part of the DR record). The format of the material to be provided in DR is expected to be standardized to make it easy for depositing and reviewing (“validating”) scientists to be sure that all the needed information is provided. When satisfactory, the DR is validated, becoming a vDR registered in the database. vDRs are made public in fully open access, either immediately, or, if the authors’ request, after an embargo period until the article is accepted. This optional embargo period is very important to ensure scientists who think their work is highly competitive and do not want their experimental ideas and results to be public before the final article is actually accepted.

Articles, in my proposal, are almost identical to what they currently are, with a key difference: they are composed of vDRs instead of the current reports with incomplete procedures and partial results. The merit of the manuscript is evaluated by reviewers, as it currently is, on the basis of the interest of the biological question addressed, and the strength and convergence of the experimental evidence to support the conclusions. Of course, for their evaluation reviewers have privileged and confidential full access to the vDRs even if under embargo. Should the reviewers request additional experiments, the authors need to have them validated independently as vDRs before resubmission to keep the same high standards throughout the publication process. Moreover, as indicated below, after article publication, a vDR remains an open record that can be enriched later by the same authors or others, always with the same high-quality requirements.

## Advantages of vDRs in addition to increased reproducibility and validity

By ensuring the full communication of all experimental material, procedures, and data, in a controlled manner, the proposed vDR procedure is expected to increase the validity of results and their reproducibility, ultimately leading to an overall gain in scientific quality and transparency. In addition, it would have many other advantages that can be summarized as follows.

A DR is one substantial set of identical or complementary experiments, and submission quickly follows their completion, before details are lost or forgotten. The persons actually involved in the experiments submit the DR and are its authors with explicit indication of their precise role. This credit attribution is easy for one set of experiments, whereas it is complex in articles that include numerous experiments and results. This can be a significant improvement in relation to the sensitive issues of authorship, contribution credit, and tractability.

Validation is factual, based only on the technical quality of the methods, data, and analyses, but unbiased by the “interest” of the question or lack of it. Moreover, the DR submission can be easily anonymized in contrast to current whole papers.

A vDR is easy to retrieve later with text-mining tools and links between databases. Anyone planning an experiment can easily access all related vDRs, in contrast to the current difficulties that can be encountered in retrieving previously published results and procedures often hidden in articles. Accessibility to vDRs will be a very important objective. This will be facilitated by a standardized shell for the report structure, easy to search, and the development of a specific openly accessible archiving and mining engine, similar to what PubMed does for published articles.

Scientists who repeat the same or a similar experiment can publish their material, methods, and results in the database where the first vDR is included, following the same high-standard validation procedure. If the results are similar to the original ones, conclusions are strengthened, whereas if they differ, they raise attention to possible uncontrolled parameters or underlying variability, prompting moderation of the conclusions. Negative results and replication failures are published and are as tractable as positive results. Their availability together with the initial results is a practical advantage. Thus, the logs of vDRs (including revisions, repetitions when available, or their failure) become invaluable open and updatable documents.

The vDRs are included in curricula vitae and facilitate the evaluation of expertise, know-how, and contributions, especially useful for students, early-career scientists, and technicians, even if the final full papers are not (yet) published or if the overall scientific program is stopped.

vDRs are fully open (after the optional embargo period) and can be used for new analysis or as supporting evidence, with explicit reference to the source, in a completely transparent manner. vDRs can be used by any scientist for different analyses or meta-analyses. This releases the full potential of open and interactive science.

The vDR databases provide an exhaustive archiving system of scientific production. To a large extent, they can, in the long-run, replace the laboratory notebooks, dematerialized or not, which are not open and not searchable, and are hindered by archiving difficulties and, in many cases, their unavoidable disappearance or loss. As long as these databases are maintained, mirrored, and saved, they are an invaluable resource, including for those who will study the history of science.

## Addressing the potential difficulties of vDRs and two-step publication

The idea of vDRs immediately raises questions/objections that can be addressed one by one.

### Who is going to validate the DRs?

Potential reviewers of DRs include all scientists who currently review manuscripts, as well as experienced postdocs, graduate students, or technicians who are experts on the methodological approach, but are currently not solicited for article reviews. One DR being focused on one type of experiment makes it easy and fast to evaluate for familiar reviewers. Statistical advisors have quick control. It is anticipated that lists of points to be checked in DRs (including the exhaustive description of materials) will progressively become standardized, making the validation process easier and faster. Such checklists of key points necessary for a DR to be valid can be easily built a priori and evolve with experience. They can be made available to facilitate the preparation of DRs.

### Is this two-step process for scientific publication going to slow down research?

Of course DR submission and validation take time. But the submission is done by the scientists who conducted the experiments, soon after their completion. The workload is spread among all those who participate in an experiment, and this reporting is supposed to be performed anyway, although in a less formal and standardized manner, in laboratory notebooks. Submitting DRs is done in parallel with other experiments, and when the time to write the article comes, materials, methods, and results are already completed and available. Minor initial slowing is largely balanced by the expected long-term time savings because of increased validity and reproducibility. Moreover, if the authors do not request an embargo, the vDRs can be immediately accessible to the scientific community as current preprints but with better validity.

### Will scientists want to submit their results to a vDR database?

Having results saved as vDRs would bring many benefits to both senior and junior scientists. vDRs are strong and tractable pieces on which publications are built, whether they are large multiauthor/laboratory studies, resulting in important “big” articles or smaller focused ones. Laboratory expertise and know-how can be appreciated based on its vDRs. For junior scientists, vDRs attest their exact contribution and expertise even when the full paper is not yet accepted or if the overall project fails and no publication is ever produced. In fact, valid results obtained in lines of research that are not pursued to the full article level can be made available and contribute to scientific knowledge. The practice of writing DRs is excellent training for experimental precision and rigor for early career scientists. A short internship can result in a vDR, and negative results of well done experiments can be deposited and appreciated. The recent spectacular success of preprint databases demonstrates that scientists are looking forward to improving publication procedures.

### Will dissociation of results from the broader context of the article alter their evaluation?

Currently, experiments reported in articles are introduced and their results interpreted in the context of a broader biological question. It can be argued that when the two are dissociated, it could lead to difficulties or lack of rigor in the evaluation of the value of the experiment and its results. In the current proposal, when DRs are submitted they first include “a clear exposure of the precise biological question that the experiment directly aims to answer.” Any scientific experiment is expected to answer a precise question, whether it is descriptive (e.g., what is the value of specific measurements within a certain sample of objects or populations? Are several parameters correlated?) or mechanistic (e.g., what is the effect of a particular perturbation on specific measurements?). This level is distinct from the scientific context in which the experiment is conducted, such as a broader question about a basic biological function at the cellular or system level, or about a disease mechanism. At the level of DRs, the validation is proposed to be performed based on simple criteria. (1) Is the precise question asked sufficiently clear to be actually answered? (2) Does the reported experiment satisfactorily allow the question to be answered (are the material and procedures appropriate?). (3) Are the results sufficiently convincing (are their quality, clarity, and analytical procedure satisfactory? Are the number of replicates sufficient and the statistical analysis correct to draw conclusions?). This type of evaluation does not require the broader context. In contrast, the scientific articles composed of vDRs are expected to put the experiments and their results in a broader meaningful context, and to gather a sufficient variety of converging evidence to bring a convincing contribution to a relevant biological problem. I would argue that the distinction between these two levels has beneficial effects by avoiding confusion between two logically different steps.

### Who is going to organize vDR databases and cover their cost?

A way to answer this question is to ask who is currently losing time and money with the insufficient quality and lack of reproducibility in biomedical sciences (Freedman et al., 2015). The primary source of funding is public money, supplemented by private foundations or bodies, including charities and generous donators (Viergever and Hendriks, 2016). To increase research quality and yield, all these funders could consider building vDR databases for the research they support and request their use. Academic institutions would clearly benefit from offering a vDR database to their members, ensuring their preeminence in high-quality and open science. The vDR databases can also replace or be combined with and improve other methods of conserving raw data and material from notebooks. Publishers and journals have a key role to play. They would boost their quality, hence their potential impact, by requesting that submitted manuscripts only include vDRs. They could naturally consider setting up vDR databases themselves. Any academic institution or funder with ambitious goals and a vision for high-quality open science could start the practice of vDR databases. Finally, the industry, which deplores the lack of reliability of academic productions and the need to repeat published experiments (Prinz et al., 2011), could be interested in supporting the initiative. Modest DR submission fees could also cover their cost. A strength of the vDR database proposal is that it can be started at a “local” level (i.e., individual funding agency or academic institution). This would increase their respectability and visibility, hopefully encouraging others to move in the same direction. The key of course is that all interested actors make sure that once vDRs are published they are accessible and searchable, and that high standards are agreed on. Poor quality will be plainly visible and counter-selected. It is expected that reliable vDR databases in contrast to poor or insufficiently stringent databases will be easily identifiable based on the quality of their content. An archiving and mining engine for all available vDR databases (a “vDR PubMed”) will be a very valuable development to facilitate their access and boost their usefulness. Finally and importantly, the requirement for vDR to publish articles in respectable journals and the open access to vDR databases should make quality checking easier, and should facilitate the fight against predatory publishers, paper mills, and fake articles.

## Conclusion

I propose dissociating scientific publication in two steps: (1) first deposit and validate substantial sets of experiments and results in ultimately open, searchable, and updatable databases ; and (2) then write articles using only deposited, validated results as strong and open building material. I argue that in biomedical sciences it has the potential to significantly improve the output quality, addressing many issues about validity, reproducibility, and openness of science, as well as providing better recognition of scientists’ contributions and long-term storage of the material on which science is built. I am not aware of similar proposals and hope this proposal will trigger interest and generate discussion and, hopefully, action.

## Synthesis

Reviewing Editor: Karl Herrup, University of Pittsburgh School of Medicine Decisions are customarily a result of the Reviewing Editor and the peer reviewers coming together and discussing their recommendations until a consensus is reached. When revisions are invited, a fact-based synthesis statement explaining their decision and outlining what is needed to prepare a revision will be listed below. The following reviewer(s) agreed to reveal their identity: Eve Marder.

The author proposes as dramatic change in the structure of scientific publications. The process would be broken down into two steps: modules that contained only detailed methods and results followed by “full” papers that introduce the scientific background of the work and its implications for the field. The motivation for this approach is the observed lack of reproducibility and replicability in the field. The proposal is a thoughtful one and merits publication as a means to stimulate discussion of this important issue, but some details of the idea need to be more fully fleshed out.

Advantages of this proposal are clear: many simple experiments now do not see the light of day because they don’t make complete stories, and many trainees fail to get credit for experiments that are not considered to be part of a potentially high profile paper.

Potential disadvantages are that in divorcing data from their interpretation, the initial scrutiny of the data may not be as rigorous or may focus on the wrong aspects of the work since the motivation for conducting the experiment can only be inferred.

The proposal seems to enable the writing of a hybrid paper - one in which the modules that end up being a part of the “full” manuscript contain DRs that were not all performed by the lab writing the paper. Full credit is given to the people who did the work that’s an advantage. The disadvantage is that the “full” paper envisioned by the original scientists might no longer be competitive.

It would help as well if as part of the discussion there were some boundaries placed on the size of “DR”. The borders will be fuzzy to be sure, but an afternoon’s work of an undergraduate student might be considered to be an experimental result simply too small or trivial to be useful. The inverse might also be true, that a large and complex experiment might not be especially useful in the newly envisioned two-step, modular structure.

As the DR’s will only be broadly useful if they are easily accessible, consideration might be given to requiring a standardized shell for the DR report structure that is easily retrievable by generic search engines. Alternatively a centrally housed PubMed-like search engine should be proposed as part of the plan.

Finally, the author may wish to compare their proposal with proposals from journals such as eLife which would allow a journal to review directly from BioRxiv.

## Author Response

Response to “Synthesis Statement for Author”:

The author proposes as dramatic change in the structure of scientific publications. The process would be broken down into two steps: modules that contained only detailed methods and results followed by “full” papers that introduce the scientific background of the work and its implications for the field. The motivation for this approach is the observed lack of reproducibility and replicability in the field. The proposal is a thoughtful one and merits publication as a means to stimulate discussion of this important issue, but some details of the idea need to be more fully fleshed out.

Advantages of this proposal are clear: many simple experiments now do not see the light of day because they don’t make complete stories, and many trainees fail to get credit for experiments that are not considered to be part of a potentially high profile paper.

Potential disadvantages are that in divorcing data from their interpretation, the initial scrutiny of the data may not be as rigorous or may focus on the wrong aspects of the work since the motivation for conducting the experiment can only be inferred.

Thank you for bringing up this important point.

In the description of the DRs, p. 7 I have modified the wording of the first element to be included in DRs: "1) a clear exposure of the precise biological question that the experiment directly aims to answer;"

I now discuss this point and related concerns in the last part of the manuscript as an additional #4 paragraph p. 12: "4) Will dissociation of results from the broader context of the article alter their evaluation?

Currently, experiments reported in articles are introduced and their results interpreted in the context of a broader biological question. It can be argued that when the two are dissociated it could lead to difficulties or lack of rigor in the evaluation of the value of the experiment and its results. In the current proposal, when DRs are submitted they first include “a clear exposure of the precise biological question that the experiment directly aims to answer”. Any scientific experiment is expected to answer a precise question, whether it is descriptive (e.g. what is the value of specific measurement(s) within a certain sample of objects or populations? Are several parameters correlated?) or mechanistic (e.g., what is the effect of a particular perturbation on specific measurements?). This level is distinct from the scientific context in which the experiment is carried out, such as a broader question about a basic biological function at the cellular or system level, or about a disease mechanism. At the level of DR the validation is proposed to be done on simple criteria: i) Is the precise question asked sufficiently clear to be actually answered? ; ii) Does the reported experiment satisfactorily allows to answer the question (are the material and procedures appropriate?); iii) Are the results sufficiently convincing (are their quality, clarity and analytical procedure satisfactory? are the number of replicates sufficient and the statistical analysis correct to draw conclusions?). This type of evaluation does not require the broader context. In contrast, the scientific articles comprised of vDRs are expected to put the experiments and their results in a broader meaningful context, and to gather a sufficient variety of converging evidence to bring a convincing contribution to a relevant biological problem. I would argue that the distinction between these two levels has beneficial effects by avoiding confusion between two logically different steps.”

The proposal seems to enable the writing of a hybrid paper - one in which the modules that end up being a part of the “full” manuscript contain DRs that were not all performed by the lab writing the paper. A clear advantage of this outcome is that full credit is given to the people who did the work. A disadvantage is that the “full” paper envisioned by the original scientists might no longer be competitive. The suggested embargo idea might need a bit more detail to deal with this consideration.

Thank you also for this comment. Indeed, such “hybrid papers” could be useful for scientific progress but somehow detrimental for scientists. I included more details on the optional embargo which is the key for the authors to protect their results if they want it, until the final article is published.

Introduction, p. 4 “they deposit their full methods and results in a “validating” database, which is open (after an embargo period if requested) and updatable. Second, later to write articles scientists use as many sets of validated deposited results (already open or still under embargo) as needed.”

Description of vDRs p. 8 end of 1st paragraph: “This optional embargo period is very important to ensure scientists who do not want their experimental ideas and results to be public before the final article is actually accepted.”

It would help as well if as part of the discussion there were some boundaries placed on the size of “DR”. The borders will be fuzzy to be sure, but an afternoon’s work of an undergraduate student might be considered to be an experimental result simply too small or trivial to be useful. The inverse might also be true, that a large and complex experiment might not be especially useful in the newly envisioned modular structure.

I fully agree with this suggestion, although it is difficult to be very specific here.

I added the word “substantial” in the description of material for DR in the Abstract, p. 2, “... when they complete a substantial coherent set of experiments” and in the Conclusion, p.14, “i) first deposit and validate substantial sets of experiments and results...”

Two sentences in the description of DRs p. 7: “Preliminary or first results cannot be considered as DR, which must correspond to a full experiment or set of related experiments with clear and conclusive (positive or negative) results. At the other end of the spectrum, a very complex set of experiments and results that can be divided into logically and methodologically separated subsets, could be deposited as several DRs.”

In the long run, the DR’s will only be broadly useful if they are easily accessible. Therefore, consideration might be given to requiring a standardized shell for the DR report structure that is easily retrievable by generic search engines. Alternatively a centrally housed PubMed-like search engine should be proposed as part of the plan.

The two points raised here are also important and not necessarily exclusive from each other. This was only suggested and I included more precise sentences.

p. 8, l. 12 : “The format of the material to be provided in DR is expected to be standardized to make it easy for depositing and reviewing (“validating”) scientists to be sure that all the needed information is provided.”

p. 9, bottom of the page: “Accessibility to vDRs will be a very important objective. This will be facilitated by a standardized shell for the report structure, easy to search, and the development of a specific openly accessible archiving and mining engine, similar to what PubMed does for published articles.”

p. 13 bottom: “An archiving and mining engine for all available vDR databases (a “vDR PubMed”) will be a very valuable development to facilitate their access and boost their usefulness.”

Finally, the author may wish to compare their proposal with proposals from journals such as eLife which would allow a journal to review directly from BioRxiv.

The interest and limitations of preprint systems were already mentioned p. 6. I have modified and completed the text to better describe the differences with the current proposal and the possible combination of vDR-based articles advantages with preprint servers. P. 6, {section sign} 2: "- Unpublished preprint publication is an important progress, whose amazing success demonstrates the possible speed of evolution in the way science is communicated. Preprint servers allow faster access to novel material and possible feedback from any reader before the work is formally submitted. Some journals, such as eLife, now accept direct submission and review of material already in BioRxiv, while others provide preprint publication service at the time of submission. These are welcome steps, which simplify the submission process. However, such preprints are just unreviewed article manuscripts with all the limitations of current articles that are discussed here. The advantages of preprint publication and the changes in articles composition proposed here (see below), will be easily combined with the publication on open databases of full articles comprising vDRs before they are actually submitted or accepted.”
